# Supplementary material for: Network extraction by routing optimization
Source: Sci Rep. 2020 Nov 30;10:20806. doi: 10.1038/s41598-020-77064-4 (PMC7704656; doi:10.1038/s41598-020-77064-4)
Supplement: Supplementary file 1 — Supplementary material 1 [file 41598_2020_77064_MOESM1_ESM.pdf]

Supplementary: "Network extraction by routing optimization."

Diego Baptista[1,+], **Daniela Leite**[1,+], **Enrico Facca**[2], **Mario Putti**[3], **and Caterina De Bacco**[1,*]

[1]Max Planck Institute for Intelligent Systems, Cyber Valley, 72076, Tübingen, Germany
[2]Centro di Ricerca Matematica Ennio De Giorgi, Scuola Normale Superiore, Piazza dei Cavalieri, 3, Pisa, Italy
[3]Department of Mathematics "Tullio Levi-Civita", University of Padua, via Trieste 63, Padua, Italy
[*]caterina.debacco@tuebingen.mpg.de
[+]These authors contributed equally to this work

S1 Examples of *graph pre-extraction* routines

We provide here several examples of networks resulting from different routing optimization setup for each of the three graph definitions.

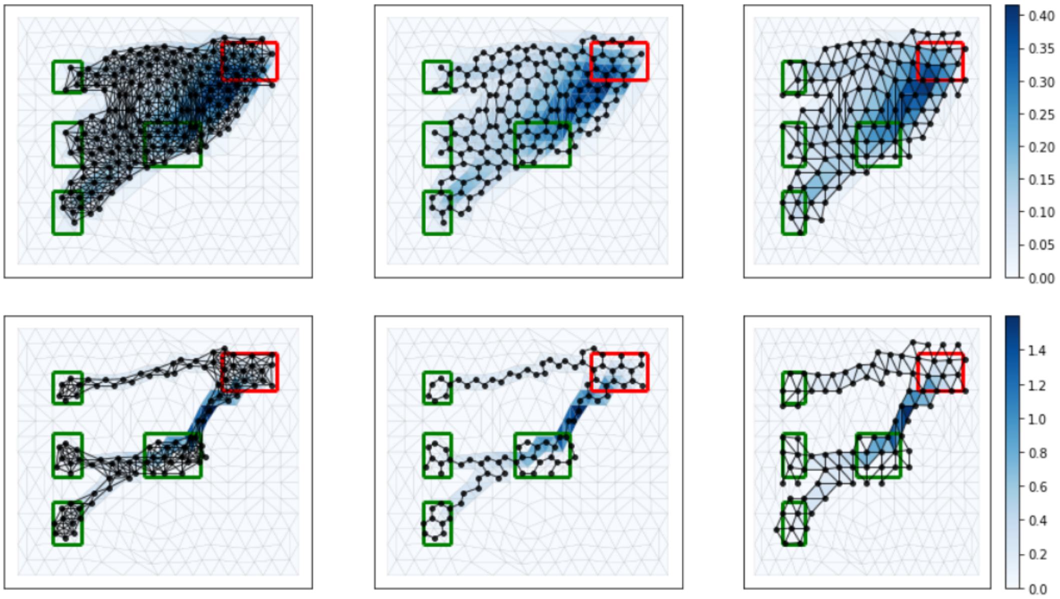

Figure S1. *Graph pre-extraction* rules for three transportation optimization problems. Top): $\beta = 1.1$. Bottom): $\beta = 1$. Left): edge-or-node sharing; center): edge-only sharing; right): original triangulation. We monitor the density μ and use parameters: $\mu_0 = 1$, $f = 5$rch, $\delta = 0.0001$, $w_{ij} = $ ER.

S2 Examples networks and routing optimization scenarios

We provide here several examples of networks resulting from different routing optimization setups for several choices of our proposed routines. This should serve as an example guideline on what parameters to choose based on the application. More specifically, we show three different setups given in input as initial routing optimization problems to the *DMK-Solver*. We consider: i) different locations of sources and sinks to show how this impacts the formation of branches and their symmetry; ii) different values of $\beta \in \{1.2, 1.3, 1.5\}$ to show how traffic consolidates into fewer edges as β grows; iii) different initial $\mu_0(x,y)$ (parabolic, delta-like and uniform) to show variability in the initial transport density.

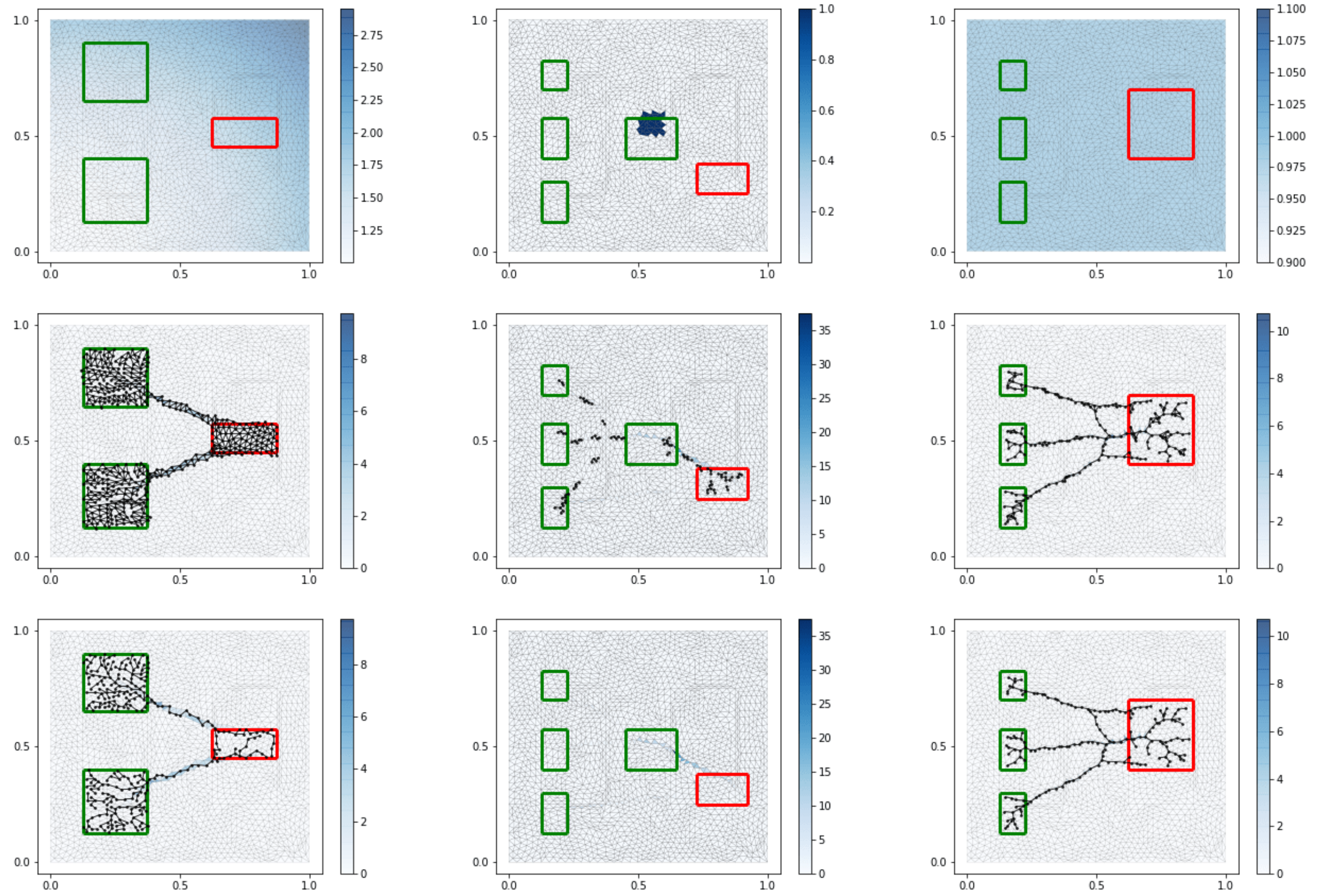

Figure S2. Network extraction examples for branched transportation optimization. Columns denote a particular setup based on source/sink locations and values of β and μ_0 as described in the sub-captions. Each row represents a step of our protocol: top) initial transport density $\mu_0(x,y)$ given in input to *DMK-Solver*; center) graph *pre-extraction* using III-AVG (left), II-AVG (center) and I-AVG (right); bottom) graph *filtering* with weight assigned with rule IBP (left and right) and no simplification (center). The color scheme refers to the value of μ. Sources and sinks are points inside the green and red rectangles, respectively. Note that pre-extraction rule II tends to break the network into disconnected components. This is also the reason why this rule scores well in terms of total path length (see Fig. 4 in the main manuscript). Since the path is already broken and sources cannot reach the sinks, it does not make sense to apply the filtering, hence we left the center-bottom plot empty.

S3 Source and sink selection

Selecting source and sink is an important task during the graph filtering step as explained in the main manuscript Sec. 3.2. Here we give examples showing why this is the case. Specifically, for a particular problem setup, we consider four cases: i) no selection of sources and sinks. This means that we simply consider sources and sinks *all* the points inside the support of the forcing function $f(x)$, input of the *DMK-Solver*. In this case, the final network structure is dominated by the many branches connecting the sources and sinks within the support of $f(x)$, thus hindering the contribution of the bulk of the network, the one connecting sources and sinks; ii) convex hull-only: we set as source (or sink) all the points inside the convex hull of the set of eligible sources (or sinks); iii) betweenness centrality-only: we set as source (or sink) all the eligible sources (or sinks) that have betweenness centrality smaller than a threshold $0 \leq \tau_{BC} \leq 1$. These two criteria miss possibly relevant sub-branches, as shown in Fig. S3 top-right and bottom-left; iv) selection using both convex-hull and betweenness centrality criteria. In this case, we capture the relevant sub-branches from both criteria, as shown in the bottom-right of Fig. S3. In the main manuscript we always consider this final criterion as it seems to capture all the relevant branches inside the support of $f(x)$. However, we encourage the final user to choose what criteria to choose based on the application. Figure S3 should help driving this decision.

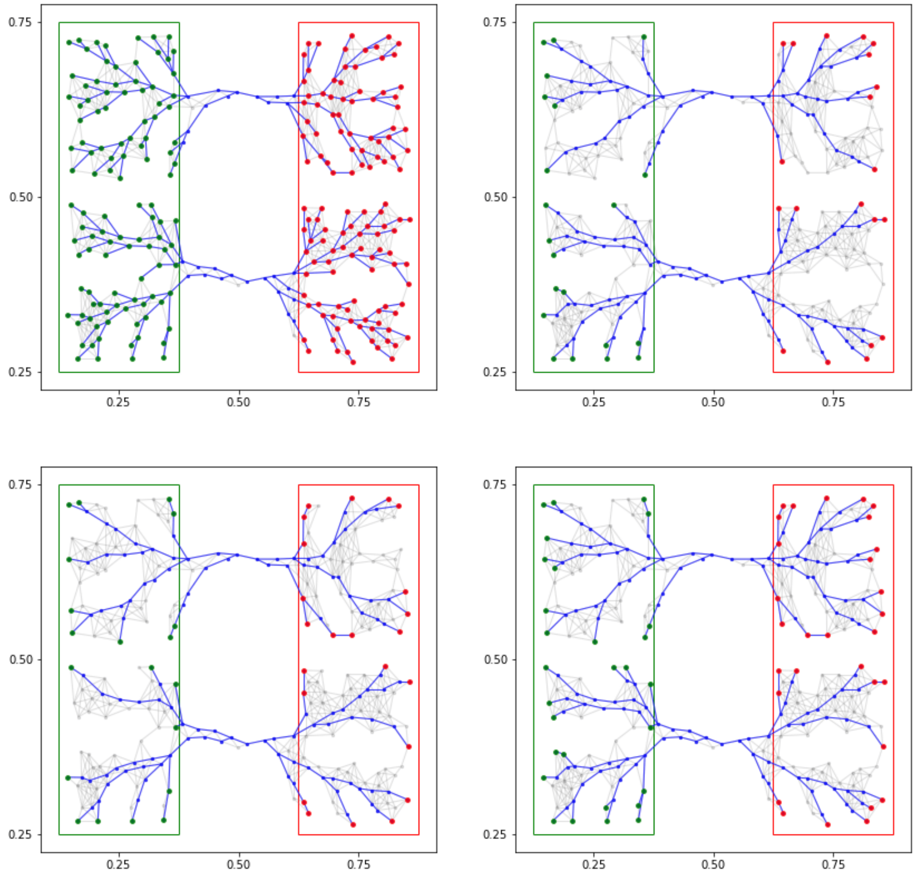

Figure S3. Choosing sources and sinks. Top-left) no selection of sources and sink ($\tau_{BC} = 1$); top-right) selection using betweenness centrality only ($\tau_{BC} = 0.01$) ; bottom-left) selection using convex hull-only ($\tau_{BC} = -1$); bottom-right) selection with convex-hull and betweenness centrality criteria ($\tau_{BC} = 0.01$).

S4 Network extraction from image

We describe here the steps followed to get the network shown in Fig. 7 in the main manuscript[1]. The original figure contains loops, however our protocol in its standard settings with filtering can only generate tree-like structures. Therefore, if we want to

[1]We used image "IMG_0379.jpg" stored at KIST_Europe_data _set/raw_images/ motion16/ inside the SMGR repository[1]. The dimension of the original one is 5184x3456 pixels. We used a 1200x1200-pixel section in Fig. 7. The coordinates of the bottom-left vertex of the box are $(2000,2000)$ (assuming $(1,1)$ to be the bottom-left pixel of the original image).

obtain a network with loops, we should consider a minor modification of our routine, as explained in detail below. In short, we need to find first a tree-like network close to the original image with loops and then give this one in input to our routine. Then, after applying our protocol, we can obtain loops by adding edges that properly *close* the loops in the tree. This can all be done in an automatized way as explained below. Otherwise, if obtaining loops is not required, our routine can be used with no modifications. The whole procedure can be divided into 6 parts: image selection and approximation, graph pre-extraction, tree reduction, terminal identification, graph filtering and edge correction.

1. Image selection and approximation: this consists in choosing an image and, if desired, mapping it into a reduced version of it. This reduction is useful when dealing with high-resolution images. We use the *resize* function included in *OpenCV*. The pixel values for the reduction image can be computed using different interpolation methods. We use linear interpolation (*cv.INTER_NEAREST*). The original image's dimensions are 1200x1200. The reduced ones are 100x100.

2. Graph pre-extraction: the RGB values of the pixels are mapped into an integer in a one-to-one way, and then they are used to define the function μ^* on each pixel. The graph G is obtained using rule I-ER as defined in Sec. 2 of the main manuscript.

3. Tree reduction: the filtering could be applied directly on G after choosing some sources and sinks, but this will generate a filtered tree-like network, i.e., no loops from the image will be captured. Thus, we propose to change G by a tree graph whose structure could then be easily "corrected" (after the filtering step) to get the mentioned loops. This tree is taken to be the *Breadth First Search* graph (G_{BFS}) of G from a random root.

4. Terminal identification: terminals (the union of sources and sinks) are defined using *filters*, i.e., squared pieces of the image. We place terminals in the graph by counting how many intersections are between the image and the boundary of the filter: if just one intersection is found, then a terminal is placed in the node with the lowest closeness centrality; if three or more intersections are found, then a terminal is placed in the node with the highest closeness centrality. We cover the whole image by disjoint filters, thus we do not miss the relevant parts of the image. Notice that if some parts of the image do not have a representative in one of the two source or sink sets, then they will not be part of the filtered graph.

5. Graph filtering: G_{BFS} is filtered as in Sec. 3 of the main manuscript (to obtain a graph G_f) by defining the source set (S^+) to be a random element on the set of terminals and the sink set (S^-) to be the remaining ones; $\beta_d = 1$ in Fig 7.

6. Edge correction: to *close* the loops we use the following rule. For each pair of terminals $u, v \in G_f$, if the shortest path P^{uv}_{BFS} on G_{BFS} is *long* but the shortest path P^{uv} on G is *short,* then add P^{uv} to G_f. The notions of *short* and *long* paths are precisely defined by introducing two parameters L_{BFS} and L_G, respectively. We say that a path P is *long*, if $l(P) > L_{BFS}$; and it is *short,* if $l(P) < L_G$, where l is the number of nodes in the path P; the values of L_{BFS} and L_G can be tuned based on the system size at hand.

References

1. Dirnberger, M., Mehlhorn, K. & Mehlhorn, T. Introducing the slime mold graph repository. *J. Phys. D: Appl. Phys.* **50**, 264001 (2017).
